# Lentiviral gene therapy reverts GPIX expression and phenotype in Bernard-Soulier syndrome type C

**DOI:** 10.1016/j.omtn.2023.08.004

**Published:** 2023-08-20

**Authors:** Raimondo De Cristofaro

**Affiliations:** 1 Dipartimento di Medicina e Chirurgia Traslazionale, Università Cattolica S. Cuore, Facoltà di Medicina e Chirurgia “Agostino Gemelli,” Roma, Italy; 2 Servizio Malattie Emorragiche e Trombotiche, Fondazione Policlinico Universitario “A. Gemell” IRCCS, Roma, Italy

Dear Editor,

Bernard-Soulier syndrome (BSS) is a bleeding disorder associated with abnormal platelets, due to mutations in one of three genes: GpIbα (BSS-type A), GpIbβ (BSS-type B), or GpIX (BSS-type C) that are assembled in a glycoprotein complex on the surface of platelet membrane.^1^ Two GPIb-IX adducts bind to one GpV molecule, forming the trimolecular GPIb-IX-V complex. The latter is the main receptor of von Willebrand factor.^2^ In addition, thrombin, the final activated factor of the coagulation cascade, can bind with high affinity to GpIbα.^3^^,^^4^ Interaction of the GPIb-IX-V complex to von Willebrand factor allows platelets to stick to the blood vessel wall at the site of the injury. These platelets form clots, plugging holes in the blood vessels to help stop bleeding. The presence of about one hundred natural mutations in GpIbα, GpIbβ, or GpIX prevents the formation or the complex’s interaction with von Willebrand factor, leading to the excessive bleeding characteristic of BSS. The therapy of this genetic disorder is usually symptomatic, based on platelet transfusion and the use of hemostatic agents such as tranexamic acid, desmopressin, oral contraceptives during menses, and recombinant FVIIa.^5^^,^^6^ More aggressive therapies, such as bone marrow transplantation, have also been employed in the most severe cases.^5^ In this problematic scenario, the study by Martinez-Navajas and colleagues represents a first pre-clinical model to correct BSS with gene therapy.^7^ This elegant study recapitulates all the possible phases of the disorder. The authors first generated induced pluripotent stem cell knockout cells deprived of GpIX as a model of BSS-type C. Thereafter, by using lentiviral-based gene therapy, they showed that it’s possible to correct the gene defect, restoring a normal platelet phenotype and function. Moreover, using lentiviral transduction or CRISPR-Cas9 technology, the authors convincingly showed that this technique can be used also in human stem cells (HSCs), demonstrating for the first time that HSCs from BSS-type C patients can be rescued *ex vivo*.^7^ Gene therapy is progressively being used to correct the natural deficiency of coagulation FVIII and FIX, responsible for hemophilia A and B, respectively.^8^^,^^9^ In these cases, gene therapy employed adeno-associated-virus (AAV) gene transfer. Lentiviral-associated gene editing and AAV gene transfer show different theoretical risk profiles but also different benefits. Most lentiviral vectors arise in fact from the human immunodeficiency virus type 1 and retain the ability to stably integrate into the genome of infected cells, allowing them to produce stable gene expression. However, lentiviral-associated gene editing would carry the potential to cause oncogenic, infectious, and other transformative changes to infected cells. At variance, the AAV-based gene therapy is epigenetic in essence, is characterized by higher biosafety, and produces transgenic proteins, whose expression, however, tends to decrease over time. Thus, once the risk profile is minimized by future studies, lentiviral-associated gene therapy could represent an actual, definitive, and efficient tool for a cure of severe inherited hemorrhagic diseases, even at the level of a single cell line, as occurring in BSS.
